# Choledochal cysts in children: How to Diagnose and Operate on

**DOI:** 10.6061/clinics/2020/e1539

**Published:** 2020-03-16

**Authors:** Ana Cristina Aoun Tannuri, Lucas Arjona de Andrade Hara, Guilherme de Freitas Paganoti, Wagner de Castro Andrade, Uenis Tannuri

**Affiliations:** Divisao de Cirurgia Pediatrica, Unidade Pediatrica de Transplante de Figado e Laboratorio de Pesquisa em Cirurgia Pediatrica (LIM 30), Faculdade de Medicina FMUSP, Universidade de Sao Paulo, Sao Paulo, SP, BR

**Keywords:** Choledochal Cyst, Pancreaticobiliary Maljunction, Pancreatitis, Congenital Biliary Dilatation, Hepaticojejunostomy

## Abstract

**OBJECTIVE:**

To identify the best mode for diagnosing and treating the patients with choledochal cysts.

**METHODS:**

A retrospective study was performed with medical records of patients diagnosed with choledochal cysts from January 1994 to December 2017. In all cases, the diagnosis was based on ultrasound examination. All the patients underwent cyst resection and were divided in two groups: bile enteric anastomosis in the high portion of the common hepatic duct or in the dilated lower portion.

**RESULTS:**

Eighty-one cases were studied. The age of presentation was 4 y 2 mo ± 4 y 1 mo, and the age for the surgical treatment was 5 y 5 mo ± 4 y 6 mo. In 61 cases, US was the only image examination performed. There were 67 cases of Todani type I (82.7%), 13 cases of type IV (16.0%) and one case of type III (1.2%). Nine patients (29.0%) in the first period and 2 patients (4.0%) in the second period presented with postoperative complications (*p*=0.016).

**CONCLUSION:**

In patients with choledochal cysts, US is the only necessary diagnostic imaging. Performing the bile enteric anastomosis in the lower portion of the common hepatic duct is safer and has a lower risk of complications.

## INTRODUCTION

Choledochal cyst, or congenital bile duct dilatation, is a rare pathology, with a higher incidence in the Far East (1,2). It was first described in 1723 by Vater and Ezler and has a predisposition to develop into cholangiocarcinoma in adult life. The etiology is not completely understood; however, the most accepted hypothesis states its association with an anomalous pancreaticobiliary junction (APBDJ), which causes pancreatic secretion reflux into the biliary ducts, causing inflammation and duct dilatation. In some studies, the prevalence of APBDJ association with choledochal cysts is 97% (3-6).

It is believed that a well performed ultrasonography (US) is the only imaging exam necessary for diagnosis prior to surgery. Other types of imaging may assist in surgical planning, but they do not change the final management of the disease.

Cyst excision and biliodigestive reconstruction by hepaticoduodenostomy or hepaticojejunostomy with Roux-en-Y are the preferred methods to manage the disease in order to resolve biliary obstruction and eliminate the possibility of malignization of the dilated portion of the biliary duct (7-11). However, performing biliodigestive anastomosis in a lower portion of the common hepatic duct, where the diameter is wider, facilitates the surgical procedure and likely reduces the occurrence of postoperative complications.

Currently, with the modern imaging methods for diagnosing biliary diseases, as well as the different types of surgical treatments for these pathologies, there is no recent study that defines the best mode for diagnosing and treating patients with choledochal cysts. Therefore, these are the main objectives of the current study based on the experience of patients in a period of 24 years treated in a tertiary surgical university pediatric center, which has additional experience in liver transplantation.

## MATERIALS AND METHODS

A retrospective study was performed with medical records from São Paulo University Medical School Children’s Institute from January 1994 to December 2017. Our research included gender, symptoms at presentation, age, medical imaging, type of dilatation (following the Todani’s classification (12)), performed surgery, complications and survival for all the patients. The study protocol was approved by the ethical Committee of our Institution.

In our service, most choledochal cyst cases were referred from primary or secondary care public medical services. Some cases were referred to our institution with prior imaging exams, such as US, magnetic resonance cholangiopancreatography (MRCP) or computed tomography (CT). In these cases, US was repeated under our care, and if the diagnosis was confirmed, surgery was recommended for the patient.

**Figure 1:**
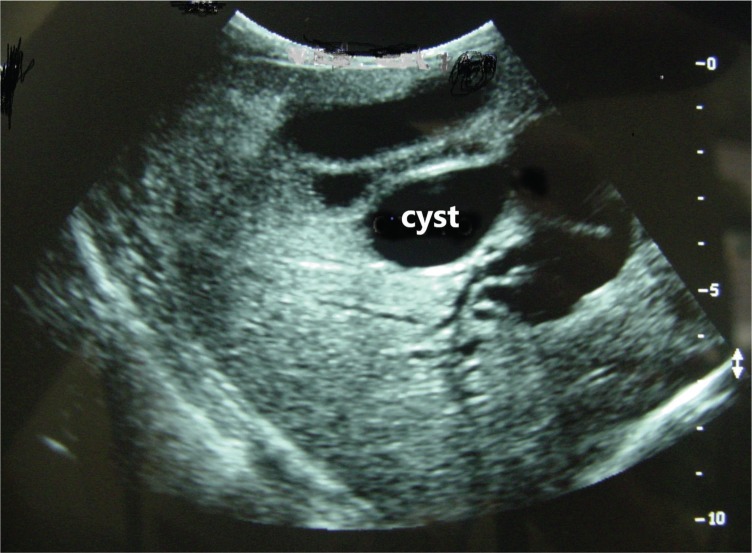
US image of choledochal cyst. Note that no other examination is necessary for surgery planning.

In cases without imaging before referral, an US examination was done and surgery was performed without additional imaging (Figure 1). As a tertiary care center, we have an experienced team of radiologists able to perform US examination in children’s biliary trees. For the statistical analysis, we only considered imaging that was performed in our institution.

**Figure 2:**
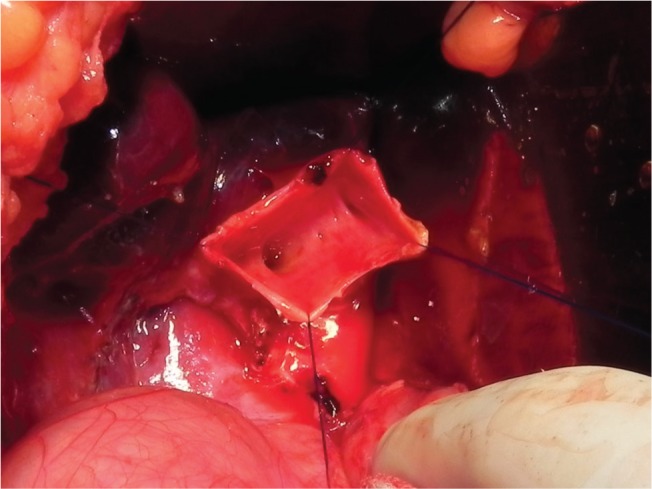
Final aspect after resection of the choledochal cyst. Note the small dilated portion of the common hepatic duct and the orifices of both hepatic ducts.

Surgery was performed in our institution using two methods. The senior surgeons (UT and ACAT) participated in all surgeries. The patients were divided into two groups according to the period and procedure. During the period of January 1994 to December 2005 (first period), a biliary reconstruction through hepaticojejunostomy Roux-en-Y was done. The anastomosis was performed in the higher portion of the common hepatic duct, leaving a narrower common hepatic duct diameter for suturing. During the period of January 2006 to December 2017 (second period), we performed a lower anastomosis at the common hepatic duct, leaving a small dilated portion of the common hepatic duct and permitting an easier anastomosis (Figure 2). In both cases, anastomosis was made with a 5.0 or 6.0 vicryl suture line, with interrupted sutures.

When it was not possible to completely excise the cyst mucosa due to adherence to another structure, such as the pancreas parenchyma, inferior vena cava or hepatic hilar vessels, Lilly’s technique was used. This technique consists of mucosal excision through cauterization, while the serosa remains attached to the adhering structures (13). Postoperative complications were studied and graded by the Clavien-Dindo classification (14). The follow-up periods varied from 18 months to 19 years.

### Statistical analysis

Numerical data were presented as the mean ± standard deviation, and categorical data were presented as frequencies and percentages. A chi-square test was applied to compare the complication frequency in the two periods of time. Statistical analysis was made using R-Commander software though R programming language. A *p*-value was considered significant if *p*<0.05.

## RESULTS

Eighty-one cases of choledochal cysts were found in our institution during the period of study. Fifty-seven patients (70.3%) were female and 24 (29.7%) were male.

The age of presentation was 4 y 2 mo ± 4 y 1 mo, and the age for the surgical treatment was 5 y 5 mo ± 4 y 6 mo, ranging from 3 days to 21 years. The most prevalent symptoms are presented in Table 1. The classic triad of the disease (jaundice, abdominal pain and palpable abdominal mass) was present in 3 cases (3.7%). The presence of at least two of the three classic symptoms was observed in 30 cases (37.0%).

Regarding the Todani’s classification, sixty-seven were type I (82.7%), thirteen were type IV (16.0%) and one was type III (1.2%).

The diagnosis was initially made by ultrasonography in all cases. In 61 cases, this was the only imaging examination performed (75.3%). To confirm the diagnosis, CT was performed, along with US, in an additional 20 cases (24.7%), and MRCP was also performed in 2 cases (2.5%). Three diagnoses (3.7%) were made by antenatal US.

In the first phase of study, an endoscopic cholangiopancreatography (ECPG) examination was used for diagnostic investigation in 7 cases (8.6%); it was diagnosed as an anomalous pancreatic biliary duct junction in 5 cases). Cholescintigraphy was used in one case (1.2%), associated with US and CT.

There were no false negative or false positive results in ultrasonography imaging in our study. In all 20 cases, the diagnosis of choledochal cysts made by US was confirmed by CT or other imaging techniques. Endoscopic cholangiopancreatography confirmed the diagnosis of the only case of type III choledochal cysts diagnosed by US. Finally, all US diagnoses were confirmed by the operatory findings.

Among the 31 patients in the first period, 9 of them (29.0%) presented with major morbidity postoperative complications, characterized by Clavien Dindo Grade III and above: four cases of late biliary stenosis (12.9%), three cases of biliary fistula (9.7%), one case of cholangitis (3.1%), one case (3.1%) of abdominal wall infection, and two deaths (6.2%) due to postoperative biliary anastomosis dehiscence and sepsis. In the second period, 50 patients were operated on with 2 complications (4.0%): one case (2.0%) of anastomotic dehiscence that was successfully corrected by reoperation and one case (2.0%) of abdominal wall infection. Among all patients of both periods, Lilly’s maneuver was used in 6 cases (7.4%). The only patient with a Todani type III cyst was treated through sphincterotomy by ECPG. All the patients with biliary stenosis had to undergo reoperation of the biliary anastomosis. Three of them remained with percutaneous dilatation of the biliary tree, although the liver function tests were normal. The incidence of complications was statistically lower in the second period (*p*=0.016).

In the late follow up, one patient of each period had to be submitted to liver transplantation due to the development of cirrhosis and hepatic insufficiency, even with adequate biliary reconstruction. Presently, both patients are doing well. Finally, no patient presented with cancer of biliary tract.

## DISCUSSION

With regards to a large series of cases of choledochal cysts in children treated in a single reference center of pediatric liver diseases and liver transplantation, some points must be discussed.

Etiology: The etiology of the choledochal cysts is not well understood. There are many theories that try to explain its pathophysiology. The most accepted theory for choledochal cysts is Babbitt’s theory, which proposes a cause and effect correlation between an anomalous pancreaticobiliary ductal junction (APBDJ) and choledochal cysts (15). The association between APBDJ and choledochal cysts ranges from 57% to 96% (16), and some studies have identified APBJD as a bad prognostic factor (17,18).

The proposed cause for the association between choledochal cysts and APBDJ is the reflux of pancreatic secretions to the biliary tree, resulting in activation of proteolytic enzymes, which are responsible for inflammation and weakening of the common bile duct wall (19). In addition, it was verified that there is a reduction of ganglion cells in choledochal cysts wall, indicating the possibility of dilatation of the common bile duct as a result of functional obstruction (20).

In the current study, we propose that cholangiography by endoscopy in order to determine if the patient has an associated APBDJ is not necessary, since it would not modify the surgical procedure. We can verify that the majority of cases (82.7%) were included in the Todani type 1 classification, which corresponds to a fusiform dilatation of the common bile duct, followed by 16.0% of cases with Todani type IV, which is defined as cystic dilatation of extrahepatic biliary ducts (associated or not with intrahepatic ducts dilatation). This observation is similar to other published series in literature (21,22).

Gender: In the current series, we verified the predilection for females (2.3 females to 1 male), similar to other previous studies, which vary from 1.6:1 to 3.7:1 (22-24). The mean ages of symptom onset and surgical treatment were comparable to those reported in other studies (19-22), although we have verified that the diagnosis has been made earlier due to the wide use of ultrasound examination in children with nonspecific abdominal pain. Regarding the type of symptoms, we found only three cases with the classic triad, which is less than those reported in the literature at 6 to 13% (23,25,26). We also saw the presence of 2 of the 3 symptoms of the triad in 28 patients (34.6%), which is less than those reported in other series at 66 to 85% (27,28). Again, we think this difference is due to the wide utilization of ultrasound examination.

Imaging: In our service, US was used in all cases as a diagnostic tool, being the only medical imaging tool in 61 cases. Due to its low cost and high availability, US constitutes the most important imaging exam for choledochal cyst investigation (29,30). We advise that US is the only necessary imaging for surgery. Other exams, such as MRCP, TC, ERCP or cholangiography do not change the therapeutic decision (cyst excision with Roux-en-Y hepaticojejunostomy).

Classically, MRCP has been considered the gold standard imaging method for the diagnosis of choledochal cysts (16). A comparative study between MRCP and CT with 14 patients subjected to both diagnostic tools demonstrated that MRCP has a better sensibility compared to CT (100% to 90.9%) (31). However, we may consider that, currently, US examination can provide an accurate diagnosis of all details in a child with choledochal cysts, including the visualization of the intrahepatic biliary tree. MRCP has the disadvantage of the necessity of general anesthesia in many children. In addition, it is important to stress that CT may be avoided in children due to nephron and hepatotoxicity produced by the utilized contrast, along with radiation exposure. Therefore, we have elected the US examination as the gold standard examination method for the diagnosis of choledochal cysts in children. The only disadvantage of the US examination is that it cannot precisely diagnose the existence of a concomitant APBDJ. However, this diagnosis does not change the management of the disease, since Roux-en-Y biliary reconstruction should be performed independently of its existence.

Prenatal diagnosis through ultrasonography was performed in three patients in the current series. Antenatal US can make the correct diagnosis in up to 15% of choledochal cyst cases (32), even though it cannot differentiate choledochal cysts from biliary atresia in a majority of cases. The importance of this finding is that it indicates an early laparotomy, with known advantages in cases of biliary atresia.

Treatment: The treatment of choice in our institution during the first period of study was a high anastomosis through Roux-en-Y hepaticojejunostomy anastomosis. In 2006, we decided to perform a lower hepaticojejunostomy anastomosis, maintaining a wider portion of the common hepatic duct, in order to technically facilitate the anastomosis and prevent postoperative complications. We performed this procedure even in the presence of a dilated common hepatic duct, despite previous recommendations from Japan to perform the anastomosis as high as possible in the hepatic duct (33,34).

The risk of biliary cancer after a Roux-en-Y hepaticojejunostomy is controversial. It is known that the cystic dilatations have a greater possibility of malignization (Todani cysts type I and IV), so its complete excision is advised (33,34). The risk of malignization after cyst excision is 0.7% to 5.4% (33,35,36). In addition, there are reports about malignization of the remaining intrapancreatic portion of the common bile duct when it is not excised (37-39). However, there is no reported risk of malignization in the fusiform dilation of the hepatic duct.

It is important to report the interesting study of Ishibashi et al. who followed up 28 patients with incomplete choledochal cyst excision and reported no cases of malignancy (40). These authors argue that biliary cancer after Roux-en-Y hepaticojejunostomy may be due to remaining cells of an already existing carcinoma in the dilated part of the biliary tract rather than *de novo* carcinoma formed after excision. In other words, the postoperative risk of biliary cancer development is biased in many studies due to an already existing carcinoma that was not detected prior to or during surgery. In another study that included 39 patients with both APBDJ and choledochal cysts, it was found a correlation between age and biliary metaplasia, which could justify the development of biliary tract cancer (41). With this in mind, we may suggest that the hepaticojejunal anastomosis should be facilitated by leaving a portion of the dilated common hepatic duct after cyst excision if the cyst cannot be excised with a satisfactory portion of the common bile duct for anastomosis.

In pediatric patients, as in our case, the chance of an already existing cancer or metaplasia of the biliary mucosa is minimal. Therefore, the risk of biliary cancer after cyst excision is probably negligible in children, even in the long term. This allows us to use part of the cyst to facilitate the anastomosis in order to provide a safer surgery, even with the possible risk of developing biliary cancer.

Finally, the discussion about the technique of biliary reconstruction with hepaticojejunostomy or hepaticoduodenostomy is not significant. It is clear that the occurrence of duodenogastric bile reflux and reflux of duodenum content to the biliary tree observed in cases of hepaticoduodenostomy is a clear disadvantage of this technique. (42-44)

Complete cyst excision of Todani I and IV cysts is not always achievable, especially when the cyst is near an important structure, such as the vena cava. In this case, Lilly’s technique may be utilized, as we performed in 7 cases (8.6%). This procedure definitely prevents injury of the pancreas or the vena cava, as well as future malignization of the remaining mucosa.

The only case of Todani type III, also known as choledochocele, was subjected to ERCP sphincterotomy without mucosa excision. It is recognized that this type of choledochal cyst has a lower probability of malignization, which has led many centers to recommend endoscopic or surgical treatment without mucosal excision, although some centers recommend cyst excision through duodenostomy (16,45-50). In fact, there are few studies following patients with choledochocele submitted to ERCP sphincterotomy. In a study from Japan, 3 of 11 patients with choledochocele developed periampullary carcinoma, which indicates that malignization is not as rare as first thought and suggests that a disjunction of the biliary and pancreatic systems though Roux-en-Y biliary reconstruction should be the standard treatment for all choledochal cyst types (51).

Complications: Twelve patients had major postoperative complications and morbidities, characterized by Clavien Dindo Grade III and above. This incidence of postoperative complications and mortality was similar to other published series (52). However, if we consider the two periods of study, we had a lower incidence of complications in the second period, when the hepatic jejunal anastomosis was performed in the lower portion of the common hepatic duct (*p*=0.016). This important conclusion is clear evidence that we must perform the biliary anastomosis according to this principle, and we must avoid anastomoses in the high portion of common hepatic duct, where the diameter is usually narrower.

Finally, two patients had to undergo liver transplantation in the late follow up, even with adequate biliary reconstruction. In these patients, biliary cirrhosis was not alleviated by adequate bile drainage, which progressed to hepatic insufficiency. Therefore, liver transplantation was necessary, despite the literature evidence of biliary cirrhosis regression after surgical treatment.

In conclusion, the current study shows that for the patients with choledochal cysts, US is the only necessary diagnostic imaging required for the final diagnosis and surgical planning. The bile-enteric anastomosis must be performed in the dilated lower portion of the common hepatic duct in order to guarantee a safe anastomosis and reduce the risks of postoperative complications.

## AUTHOR CONTRIBUTIONS

Tannuri ACA was responsible for the conception and design of the work, acquisition, analysis and interpretation of data. Hara LAA, Paganoti GF, Andrade WC designed the study, were responsible for the acquisition, analysis and interpretation of data, revision for important intellectual content and final approval of manuscript version to be published, agreement to be accountable for all aspects of the work in ensuring that questions related to the accuracy or integrity of any part of the work are appropriately investigated and resolved. Tannuri U was responsible for the conception of the work, analysis, and interpretation of data, revision for important intellectual content and final approval of the manuscript version to be published, agreement to be accountable for all aspects of the work in ensuring that questions related to the accuracy or integrity of any part of the work are appropriately investigated and resolved.

## Figures and Tables

**Table 1:** Symptoms presented by the patients with choledochal cyst.

Symptom	Number of patients (%)
abdominal pain	48 (59.2%)
jaundice	45 (55.5%)
episodes of pancreatitis	13 (16.0%)
palpable abdominal mass	13 (16.0%)
vomits	12 (14.8%)
fecal acholia	8 (9.9%)
choluria	8 (9.9%)
asymptomatic	5 (6.2%)
